# Transcriptome profiling in depression with and without loss of appetite

**DOI:** 10.1192/j.eurpsy.2023.910

**Published:** 2023-07-19

**Authors:** J. Pawlak, A. Szczepankiewicz, K. Bilska, P. Kapelski, P. Zakowicz, E. Paszynska, M. Dmitrzak-Weglarz

**Affiliations:** 1 Department of Psychiatric Genetics; 2 Molecular and Cell Biology Unit, Poznan University of Medical Sciences, Poznan; 3 Center for Child and Adolescent Treatment in Zabor, Zielona Gora; 4 Department of Integrated Dentistry, Poznan University of Medical Sciences, Poznan, Poland

## Abstract

**Introduction:**

Depression has been described very comprehensively and is a highly prevalent mental condition. However, how its features develop and clinical course shape remains not fully understood.

**Objectives:**

The study aimed to compare mRNA characteristics between specific symptoms and identification of differently expressed genes (DEGs) in patients with depression with specifiers such as loss of appetite, loss of weight, sleep disturbances and psychomotor retardation.

**Methods:**

Material and method we used was transcriptome profiling of peripheral blood mononuclear cells in 30 patients diagnosed with depressive episode in course bipolar or unipolar affective disorder. The blood samples were drawn during acute depressive episode with at least moderate severity. The diagnosis and specific symptoms were described according to ICD-10 and DSM5 criteria using SCID-I, OPCRIT, and HDRS. Agilent microarrays were used for transcriptome profiling and GeneSpring software was applied. Minimal fold change 2 and significant p-value <0.05 were assumed. DAVID and KEGG databases were searched.

**Results:**

Comparing depressed patients with and without decreased appetite or weight loss revealed 718 DEGs. When compared depressed patients with and without psychomotor retardation, 95 genes were up- or down regulated. In both comparisons DEGs were not identified as significant according to DAVID and KEGG database. When considering weight loss of more than 2 kg per month, 418 DEGs were identified. According to searched databases only one, characterized with phosphoserine phosphatase activity, was indicated as having a significant role in molecular functioning. The most numerous list of DEGs (n=855) was found when compared depressed patients with and without insomnia. Among these genes, several were indicated as significant for biological processes and cellular components: those linked with response to oxygen-compound, cytoplasmic and secretory vesicles and granules and circulatory system.

**Conclusions:**

Numerous genes are differently expressed in depression with specific clinical features, such as appetite and sleeping disturbances, but their role in pathology remains unclear. One might expect that secretory and circulation activity is involved.

This research was funded by the National Science Center, Poland (Grant No: 2016/23/B/NZ5/02634) and supported by the Poznan University of Medical Sciences in Poland (Statute sources: 502-20-22196440).

**Disclosure of Interest:**

None Declared

